# Implementation and Outcomes of an Academic Peer Coaching Program for Pediatric Residents

**DOI:** 10.7759/cureus.59846

**Published:** 2024-05-07

**Authors:** Kristin Sundy-Boyles, Kelsey Jackson, Timothy Pian, Jason Benedict, Alexis Barnes, Charles Redman, Rena Kasick

**Affiliations:** 1 Hospital Medicine, University of North Carolina at Chapel Hill School of Medicine, Chapel Hill, USA; 2 Hospital Medicine, Nationwide Children’s Hospital, Columbus, USA; 3 Pediatric Critical Care Medicine, Children's Hospital Los Angeles, Los Angeles, USA; 4 Biostatistics, The Ohio State University College of Medicine, Columbus, USA; 5 Cardiology, University of Pittsburgh Medical Center, Pittsburgh, USA; 6 Hospital Medicine, The Ohio State University College of Medicine, Columbus, USA

**Keywords:** pediatric residency, struggling learner, self-directed learning, peer mentor, clinical coaching

## Abstract

Introduction

Academic coaching fosters self-directed learning and is growing in popularity within residency programs. Implementation is often limited by available faculty time and funding. Peer coaching is an emerging alternative but is not well studied. This study aims to demonstrate the acceptability, feasibility, and efficacy of a resident peer coaching program.

Methodology

In the 2021-2022 academic year, within a large pediatric residency program, we selected and trained senior residents as coaches and interns who opted in as coachees. Coaching dyads began meeting in the fall and worked toward individualized goals throughout the year; control interns participated in routine didactics. Outcomes included Accreditation Council for Graduate Medical Education (ACGME) milestone scores and a self-assessment survey (SAS).

Results

We enrolled 15/42 (36%) interns as coachees, with the remaining 27 (64%) as controls. Narrative feedback from coaches and coachees was overall positive, and time commitment was feasible for program staff (10-12 hours/month), coaches (three to four hours/month), and coachees (one to two hours/month) with minimal financial needs. Post-intervention, more coachees than controls scored ≥4.0 on ACGME milestones systems-based practice 3 (SBP3; 3/15, 20%, vs. 2/27, 7%), SBP4 (4/15, 27%, vs. 5/27, 19%), and practice-based learning and improvement 1 (4/15, 27%, vs. 3/27, 11%). SAS response rate was 8/15 (53%) for coachees and 5/27 (19%) for controls. More coachees than controls reported baseline difficulty with time management *often* (3/8, 38%, vs. 1/5, 20%); only coachees improved post-intervention, with 0/8 (0%) having difficulty *often* versus 2/5 (40%) of controls.

Conclusions

Resident peer coaching is acceptable and feasible to implement. Coachees reported more improvement in time management than controls, and ACGME milestone scores suggest improved use of evidence-based medicine and interprofessional care coordination among coachees.

## Introduction

Academic coaching is a well-accepted model for the development of self-directed learners but is often a time- and funding-intensive endeavor at odds with the many demands on faculty clinician educators [1]. Existing literature focuses on hierarchical coaching, faculty coaching trainees, and faculty-level peer coaching programs; little is known about the outcomes of resident-level peer coaching [1-7]. Given faculty time constraints and the importance of individualized education for residents, we must better understand the feasibility and outcomes of resident-level peer coaching [8].

Unlike mentoring, which relies on expert guidance, coaching facilitates self-determination and self-awareness in coachees, promoting the skills of Master Adaptive Learners [9]. Early studies of hierarchical coaching in medicine demonstrate benefits. One program showed increased frequency and quality of faculty feedback and improved coachee communication skills [2-3]. Other coaching programs advanced residents’ use of evidence-based medicine (EBM) helped them set high-quality goals and decreased burnout in female residents [4-7]. However, successful faculty coaching programs are often supported by funded faculty time [3,10-12]. Without adequate support, the feasibility and generalizability of faculty coaching for residents is limited.

Examples of peer coaching are less common. Among faculty, peer coaching improves confidence by giving feedback and identification of maladaptive teaching habits [13-14]. Resident-level peer coaching programs in surgical residencies improve operative skills, and in an internal medicine residency, near-peer coaching improves perceived leadership skills [15-16]. In the latter example, most coachees reported preferring near-peer coaching to faculty coaching [16]. Notably, these programs did not require funding. While these studies suggest resident-level peer coaching has merit, they do not examine its application to clinical competencies beyond procedural skills. Resident-level peer coaching offers an opportunity to supplement residents’ individualized education while fostering the development of resident coaches.

This study aimed to demonstrate the acceptability and feasibility of a resident-level academic peer coaching program for pediatric residents. Secondarily, we aimed to examine the efficacy of this program by comparing pre- and post-intervention pediatric Accreditation Council for Graduate Medical Education (ACGME) milestone scores and self-assessed skills of coachees to those of control interns.

Results of this study were previously presented as abstracts at the Association of Pediatric Program Directors Spring Meeting in May 2022 and March 2023 and at Pediatric Hospital Medicine in August 2023.

## Materials and methods

Setting and participants

We implemented this peer coaching intervention during the 2021-2022 academic year within a large pediatric residency program that matriculates 37 categorical pediatric and six combined program residents (child neurology and pediatric genetics) per year. We recruited post-graduate year (PGY)-3 and PGY-4 categorical and combined program residents to be peer coaches. PGY-1 categorical and combined program residents served as the study participants in the control and intervention (coachee) groups. Peer coaching program staff included a pediatric chief resident, a residency associate program director, and an internal medicine-pediatric hospitalist with expertise in clinical coaching. This group designed and implemented the coaching program and provided training for peer coaches. Coaching interventions took place both in the clinical learning environment and outside of working hours.

Coachees opted into the coaching program. For recruitment, we described the program to residents and residency program leadership, disseminated a link to our enrollment form, and advertised the program via email. We encouraged program directors to discuss peer coaching with interns during semi-annual reviews.

Coaching intervention

Coachees participated in peer coaching in addition to routine program didactics, which consisted of case-based noon conferences three times per week, grand rounds, and two hours of intern-specific didactics during an academic half-day.

**Figure 1:**
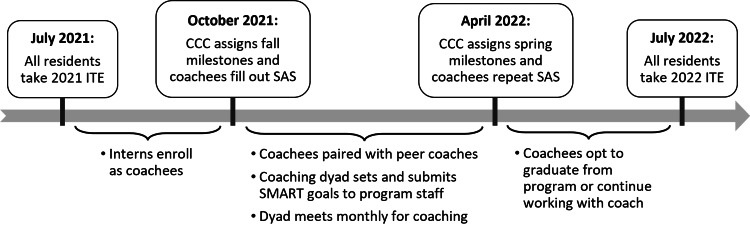
Timeline of coachee enrollment and assessment. Image credit: Kristin Sundy-Boyles. ITE, in-training exam; CCC, clinical competency committee; SAS, self-assessment survey; SMART, specific, measurable, achievable, relevant, and time-bound

Following enrollment into the peer coaching program, the coaching program staff paired the coachee with a senior resident coach. Coachees filled out a self-assessment survey (SAS) to identify and categorize their concerns (Appendix). We used the framework of self-directed learning to inform program expectations, beginning with coachees identifying knowledge and performance gaps and setting SMART goals (specific, measurable, achievable, relevant, and time-bound) with their peer coaches [17]. Coaching dyads were encouraged to meet at least monthly in a setting of the coachee’s choice and submit brief updates to program staff denoting progress or achievement of goals (Figure 1). Coachees graduated from the program when they had achieved their goals.

Control group

Control interns participated in routine program didactics but did not have a peer coach. We did not monitor other coaching or mentoring relationships during the period of study.

Coach selection and training

We selected peer coaches based on interest and clinical performance. We developed and delivered original content for coach training, including an orientation introducing clinical coaching and four evening seminars throughout the year. These educational sessions included diagnosing struggling learners, setting SMART goals, delivering effective feedback, providing motivational coaching, and introducing teaching clinical reasoning. When on elective rotations, coaches were excused from two half-days of clinical responsibilities per month to work with coachees to incentivize participation without compromising limited free time.

Measurement of outcomes

Coaches and coachees provided narrative feedback on various aspects of the coaching program. We assessed control and coachee performance pre- and post-coaching intervention using the ACGME milestone scores and SAS responses from fall 2021 and spring 2022 [18]. At our institution, milestone scores are assigned biannually by a clinical competency committee comprised of residency program leadership and core faculty, based on the summation of numerical and narrative evaluations submitted by clinical preceptors. We adapted the SAS from the semi-structured interview published by Guerrasio et al., which prompts discussion about areas of potential deficit for medical learners, including clinical skills, clinical reasoning, time management, organization, and communication [19]. We modified Guerrasio et al.’s questionnaire into a Likert scale survey, asking for the frequency of difficulty in a specific performance domain. We do not have valid evidence for this survey (Appendix). Data were collected and managed using Research Electronic Data Capture (REDCap) tools hosted at Nationwide Children’s Hospital [20-21].

Analysis of outcomes

We compared mean ACGME milestone scores and SAS responses for coachee and control groups to examine improvement from pre- to post-intervention. Given the small sample size and inherent bias of self-selected participant groups, we did not perform statistical analysis for significance. The Institutional Review Board at Nationwide Children's Hospital issued approval for this study (STUDY00002142).

## Results

We selected five PGY-3 pediatric and three PGY-4 combined program residents as peer coaches and collected data on 36/37 (97%) categorical pediatric and 6/6 (100%) combined program interns. Fifteen (36%) interns participated in peer coaching, and 27 (64%) control interns participated in routine program didactics.

Acceptability and feasibility

All eight (100%) coaches submitted feedback on the program, describing appreciation for the coaching seminars and the appropriateness of the time commitment. Four (27%) coachees submitted feedback and referred positively to the flexibility of the program but reported a lack of clarity on the peer coach role. Both groups recommended better expectation setting. Coaches described appropriate time commitment (Table 1).

**Table 1:** Selected feedback from coaches (n = 8) and coachees (n = 4).

Coach feedback	
How satisfied were you with the goals and expectations of you as a peer coach for this program? Please explain.	“I liked that we didn't have a strict deadline. I liked that I was in the same track as my mentee.”
	“I think the roles could have been a little better defined, but of course it is just the pilot! Also it was good in the fact that it was open to be malleable to whatever people needed!”
	“Was very excited to be a part of this program. I feel the explicit coaching of clinical skills is something that can be lacking in residency, so I was excited for the goals of this program and its coaches. I felt expectations in terms of time commitment and skill development were reasonable.”
How satisfied were you with the structure/flexibility of this program? Please explain.	“I think it was great to have us meet in person initially then be available via phone call/text after.”
	“Appreciated this aspect of the program. My two coachees had different needs and goals, so I was able to adapt timing and setting of coaching to them. I did not feel overburdened by demands on my time, though I did have two learners who did not require intensive intervention.”
	“Could have been a little more structure as one of my learners I only met with once despite reaching out a few times, but I really did enjoy the flexibility!”
How satisfied were you with the mini-workshop meetings? Please explain.	“Very, very helpful! could have even had one, one hour meeting once a month!”
	“Enjoyed all of the workshops and felt they were appropriate timing and very applicable”
	“Felt the debriefs and skills sessions were valuable and applicable. Would appreciate more instruction on the coaching itself, as I felt this was an area where I was more challenged as a coach.”
Please provide feedback on your overall experience participating as a peer coach in this program and/or suggestions to improve this program.	“Great experience overall as a coach. I hope I was able to help coachees, and I learned a lot about teaching and mentoring through my role as a coach. I think a next big step will be to work on the "culture of coaching," to make this a more common, visible, and accepted part of residency.”
	“I really enjoyed this experience. My mentees slowly approached their goals which was fun to see! The best part was building relationships with co-residents and establishing a community”
	“This was such a phenomenal experience - I learned SO MUCH about education and my own strengths/limitations as a peer coach.”
Coachee feedback	
Did the peer coaching program meet your goals/needs? Please explain.	“Had regular meetings with my peer coach and worked on the advice/tips she gave me. Continued to refine my goals to better meet my level of training/advancement.”
	“The coach was great and generous with their time. I felt much of the advice was not really applicable and a lot of my struggles were related to a period of high inpatient volumes and normal intern adjustment. It was helpful to hear the advice that was offered and realize I was doing the things within my power to be more efficient.”
	“Felt that in the end, I was getting most of my coaching from other seniors by seeking active feedback vs. from my peer coach.”
What did you like the most about the peer coaching program?	“It was nice to have the perspective of someone outside my immediate clinical team.”
	“Meeting a peer coach, becoming friends, setting goals helped me identify areas I wanted to work on and seek feedback on.”
	“I liked that it could be customized to fit my needs and my schedule. It was a very low-pressure way to check in with a mentor and learn through their experience how to be more efficient.”
What would you like to see improved for our peer coaching program?	“It might help to make it clear to both parties that they do not need to continue meeting if the initial goals have been met.”
	“Maybe allow peer coach to obtain (if consented by participant) feedback from seniors working with mentee so that they can continue to address and work on areas for improvement.”
	“No areas of improvement! Loved the program and the support!"

Regarding feasibility, time commitment for program staff was consistent across the design and implementation phases (10-12 hours/month). In the design phase, staff spent this time discussing program protocols and engaging stakeholders; during implementation, recruiting participants, selecting coaches, facilitating coach training, and following coaching dyads. Coaches spent their time attending coach training, preparing for coaching sessions, and meeting with coachees (three to four hours/month), and coachees were responsible for attending their coaching sessions (one to two hours/month). The residency program provided funding for dinners for four coach training sessions; there were no additional financial costs.

ACGME milestone scores

**Figure 2:**
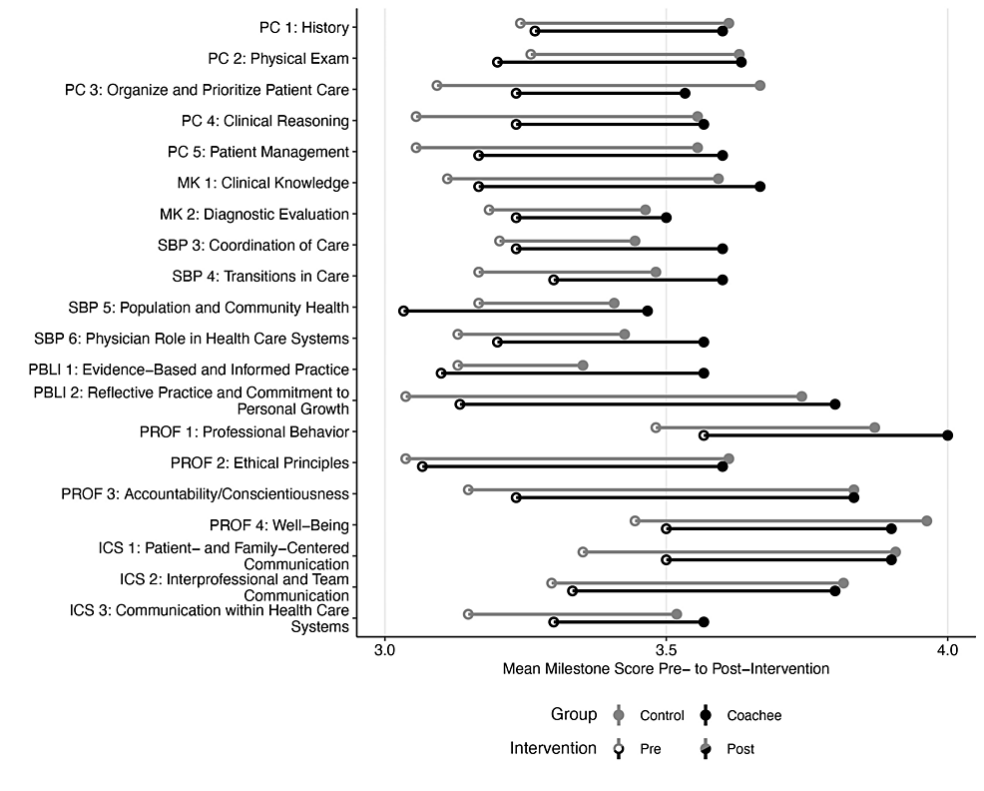
ACGME milestone scores pre- and post-coaching intervention (control, n = 27; coachee, n = 15). ACGME, Accreditation Council for Graduate Medical Education; PC, patient care; MK, medical knowledge; SBP, systems-based practice; PBLI; practice-based learning and improvement; PROF, professionalism; ICS, interpersonal and communication skills.

We examined pre- and post-intervention ACGME milestone scores for the 42 interns [18] (Figure 2). Pre-intervention scores were similar between groups, with slightly higher scores for coachees than controls in patient care (PC), systems-based practice (SBP), and interpersonal and communication skills (ICS).

Post-intervention milestone scores became more similar between groups in PC and ICS, with new variability in Practice-Based Learning and Improvement (PBLI) and Professionalism. Coachees outperformed controls on SBP3 (3/15, 20% coachees, vs. 2/27, 7% controls scored ≥4.0) and SBP4 (4/15, 27% vs. 5/27, 19% scored ≥4.0). Similarly, coachees’ PBLI1 scores were higher than those of controls (4/15, 27%, vs. 3/27, 11% scored ≥4.0). Coachees scored lower than controls on Professionalism 3 (8/15, 53%, vs. 25/27, 93% scored ≥4.0) and ICS3 (2/15, 14%, vs. 7/27, 26% scored ≥4.0). While coachees had higher PC3 scores than controls in the fall (9/15, 60%, vs. 11/27, 41% scored ≥3.5), coachees did not show the same degree of improvement as controls by the spring (2/15, 13%, vs. 12/27, 44% scored ≥4.0).

Self-assessment survey

The response rate for both the fall and spring SAS was 8/15 (53%) for coachees and 5/27 (19%) for controls (Table 2). Coachees reported more difficulty with most tasks than controls in the fall. Both groups identified difficulty with time management and reported having to arrive early or leave late from work at baseline (3/8, 38%, coachees vs. 1/5, 20% controls *often* or *all the time*). Only coachees reported improvement in this skill, and control interns reported more difficulty at follow-up (0/8, 0%, vs. 2/5, 40%, had to arrive early at or leave late from work).

**Table 2:** Self-assessment survey responses pre- and post-coaching intervention (control, n = 5; coachee, n = 8). SAS, self-assessment survey.

		Pre-coaching SAS score, *n* (%)					Post-coaching SAS score, *n* (%)				
		Never	Rarely	Sometimes	Often	All the time	Never	Rarely	Sometimes	Often	All the time
1. I have difficulty performing physical exam maneuvers.	Control	0 (0)	4 (80)	1 (20)	0 (0)	0 (0)	0 (0)	4 (80)	1 (20)	0 (0)	0 (0)
	Coachee	1 (13)	4 (50)	3 (38)	0 (0)	0 (0)	0 (0)	6 (75)	2 (25)	0 (0)	0 (0)
2. I have difficulty performing procedures.	Control	0 (0)	1 (20)	4 (80)	0 (0)	0 (0)	0 (0)	2 (40)	3 (60)	0 (0)	0 (0)
	Coachee	0 (0)	1 (13)	6 (75)	1 (13)	0 (0)	0 (0)	4 (50)	4 (50)	0 (0)	0 (0)
3. I struggle to apply my medical knowledge and use clinical reasoning.	Control	0 (0)	3 (60)	2 (40)	0 (0)	0 (0)	1 (20)	4 (80)	0 (0)	0 (0)	0 (0)
	Coachee	0 (0)	3 (38)	3 (38)	2 (25)	0 (0)	0 (0)	5 (63)	3 (38)	0 (0)	0 (0)
4. It is difficult to develop ongoing management plans for patients with multiple problems.	Control	0 (0)	0 (0)	4 (80)	1 (20)	0 (0)	1 (20)	2 (40)	2 (40)	0 (0)	0 (0)
	Coachee	0 (0)	0 (0)	7 (88)	1 (13)	0 (0)	0 (0)	2 (25)	6 (75)	0 (0)	0 (0)
5. I have a hard time narrowing my differential based on exam and lab results.	Control	0 (0)	3 (60)	2 (40)	0 (0)	0 (0)	1 (20)	4 (80)	0 (0)	0 (0)	0 (0)
	Coachee	0 (0)	4 (50)	4 (50)	0 (0)	0 (0)	0 (0)	6 (75)	2 (25)	0 (0)	0 (0)
6. I have to arrive to work early and/or leave late to finish my work.	Control	0 (0)	2 (40)	2 (40)	1 (20)	0 (0)	1 (20)	2 (40)	0 (0)	2 (40)	0 (0)
	Coachee	0 (0)	0 (0)	5 (63)	3 (38)	0 (0)	0 (0)	3 (38)	5 (63)	0 (0)	0 (0)
7. I have a system for getting through all my tasks that works for me.	Control	0 (0)	0 (0)	2 (40)	3 (60)	0 (0)	0 (0)	0 (0)	0 (0)	4 (80)	1 (20)
	Coachee	0 (0)	2 (25)	5 (63)	1 (13)	0 (0)	0 (0)	0 (0)	2 (25)	5 (63)	1 (13)
8. Sometimes it is difficult to get along with the other members of my medical team.	Control	3 (60)	2 (40)	0 (0)	0 (0)	0 (0)	3 (60)	2 (40)	0 (0)	0 (0)	0 (0)
	Coachee	6 (75)	2 (25)	0 (0)	0 (0)	0 (0)	5 (63)	3 (38)	0 (0)	0 (0)	0 (0)
9. I tend to be late to work, meetings or can sometimes forget about other scheduled obligations.	Control	2 (40)	3 (60)	0 (0)	0 (0)	0 (0)	2 (40)	3 (60)	0 (0)	0 (0)	0 (0)
	Coachee	7 (88)	1 (13)	0 (0)	0 (0)	0 (0)	6 (75)	2 (25)	0 (0)	0 (0)	0 (0)
10. I struggle with communication skills.	Control	3 (60)	2 (40)	0 (0)	0 (0)	0 (0)	0 (0)	5 (100)	0 (0)	0 (0)	0 (0)
	Coachee	2 (25)	5 (63)	1 (13)	0 (0)	0 (0)	5 (63)	3 (38)	0 (0)	0 (0)	0 (0)
11. I have difficulty receiving feedback.	Control	1 (20)	4 (80)	0 (0)	0 (0)	0 (0)	3 (60)	2 (40)	0 (0)	0 (0)	0 (0)
	Coachee	1 (13)	6 (75)	1 (13)	0 (0)	0 (0)	5 (63)	1 (13)	2 (25)	0 (0)	0 (0)
12. I notice when I struggle with certain tasks and know how to seek resources for help to get better.	Control	0 (0)	1 (20)	3 (60)	1 (20)	0 (0)	0 (0)	0 (0)	1 (20)	4 (80)	0 (0)
	Coachee	0 (0)	1 (13)	2 (25)	5 (63)	0 (0)	0 (0)	0 (0)	2 (25)	5 (63)	1 (13)

Both groups described improvement in having an organizational system, though coachees had more difficulty than controls both pre-intervention (1/8, 13%, vs. 3/5 60% *often* or *all the time*) and post-intervention (6/8, 75%, vs. 5/5, 100%). The only area in which coachees reported less difficulty than controls was with schedule management, which was consistent both pre-intervention (7/8, 88%, vs. 2/5, 40%, tend to be late or forget scheduled obligations *never*) and post-intervention (6/8, 75%, vs. 2/5, 40%).

## Discussion

This study demonstrates the acceptability and feasibility of implementing a resident-level peer coaching program in a large pediatric residency program. Limited efficacy outcomes suggest that residents who opt-in to peer coaching perceive a benefit in their time management skills and may have improved use of EBM and interprofessional care coordination skills.

Our coachees described acceptability but asked for clarity with program expectations, while coaches submitted positive narrative feedback. This is consistent with previous work, showing that peer coaching is valuable for the coachee, but even more so for the coach [14]. Coachees in our program reported appreciation of the low-pressure environment, adding to prior data that residents may prefer coaching from peers or near-peers, perhaps due to the psychological safety of a non-evaluative relationship [16]. From a feasibility standpoint, the inherent flexibility of resident elective schedules allowed us to grant coaches *protected time* for coaching in a way that could not be done for faculty. In future iterations, we expect that most coach and coachee responsibilities could be built into elective time to further minimize the additional time commitment of these resident peer coaching roles.

Areas in which coachees outperformed controls post-intervention focused on team management, interprofessional care coordination, patient hand-off, and use of EBM. This aligns with one example of coaching that saw improvement in EBM use, but we have not seen this effect on systems-based skills previously documented [7].

The SAS results are difficult to interpret due to the low response rate, but the available responses support our hypothesis that resident peer coaching improves the time management and organizational skills of coachees. Surprisingly, coachees self-assessed as having more difficulty than controls with most skills at baseline despite having similar, if not higher, milestone scores. We believe that this may be related to the self-selection process for coachee enrollment and that we may have unintentionally recruited interns with lower confidence and/or greater preexisting self-directed learning skills.

There are several limitations to this study. One is the lack of reliability and validity data for the SAS. This survey is based on expert opinion, but there are no published data on the results of the semi-structured interview [19]. We examined coachee clinical performance using ACGME milestones, however, we acknowledge that these do not have valid data for evaluation of a coaching program and do not provide a comprehensive evaluation of our program. The self-selection of control and intervention groups also limits our ability to test for statistical significance, and small sample size limits statistical power.

We consider our program’s feasibility and acceptability to be generalizable to other training programs, pediatric and otherwise, due to its use of existing leadership roles, reasonable time commitment, and minimal reliance on funding. As a next step, we plan to revise the coachee enrollment process to control for any inherent differences between residents who choose to participate in coaching and those who do not. Potential future outcomes will include evaluating documented work hours to assess time management and considering qualitative assessment of responses to the semistructured interview [19]. 

## Conclusions

We describe the design, implementation, and preliminary evaluation of a pediatric resident academic peer coaching program meant to enhance resident skills. While significance is limited by the small sample size and the self-selected intervention group, our results suggest that resident peer coaching is acceptable to coaches and coachees and is feasible to implement within a large pediatric residency program. Coachees describe greater improvement in time management than controls, and ACGME milestone scores show increased use of EBM and improved interprofessional care coordination among coachees.

## Figures and Tables

**Figure 3:**
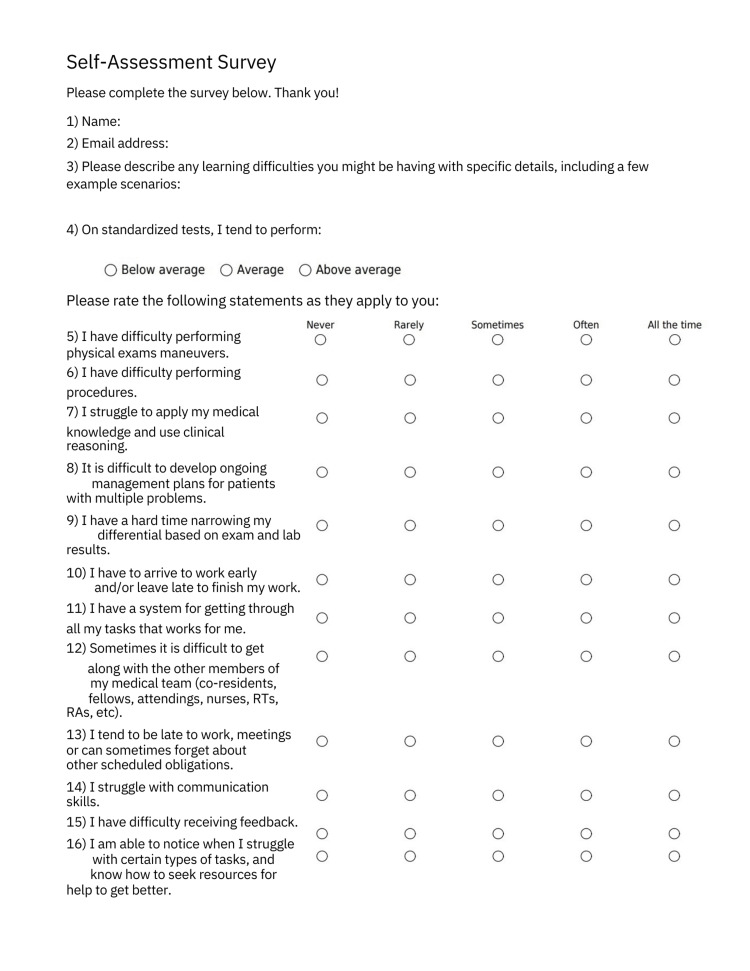
Self-assessment survey.

## Appendices

 Appendix
